# Exploring Web-Based Twitter Conversations Surrounding National Healthcare Decisions Day and Advance Care Planning From a Sociocultural Perspective: Computational Mixed Methods Analysis

**DOI:** 10.2196/35795

**Published:** 2022-04-13

**Authors:** Tahleen A Lattimer, Kelly E Tenzek, Yotam Ophir, Suzanne S Sullivan

**Affiliations:** 1 Department of Communication University at Buffalo, SUNY East Amherst, NY United States; 2 School of Nursing University at Buffalo, SUNY East Amherst, NY United States

**Keywords:** advance care planning, National Healthcare Decisions Day, Twitter, good death, hashtag activism, topic modeling, social media, end of life

## Abstract

**Background:**

Within the cultures and societies of the United States, topics related to death and dying continue to be taboo, and as a result, opportunities for presence and engagement during the end of life, which could lead to a *good death*, are avoided. Several efforts have been made to help people engage in advance care planning (ACP) conversations, including completing advance care directives so that they may express their goals of care if they become too sick to communicate their wishes. A major effort in the United States toward encouraging such challenging discussions is the annual celebration of the National Healthcare Decisions Day.

**Objective:**

This study aimed to explore ACP from a sociocultural perspective by using Twitter as a communication tool.

**Methods:**

All publicly available tweets published between August 1, 2020, and July 30, 2021 (N=9713) were collected and analyzed using the computational mixed methods Analysis of Topic Model Network approach.

**Results:**

The results revealed that conversations driven primarily by laypersons (7107/7410, 95.91% of tweets originated from unverified accounts) surrounded the following three major themes: *importance and promotion*, *surrounding language*, and *systemic issues*.

**Conclusions:**

On the basis of the results, we argue that there is a need for awareness of the barriers that people may face when engaging in ACP conversations, including systemic barriers, literacy levels, misinformation, policies (including Medicare reimbursements), and trust among health care professionals, in the United States. This is incredibly important for clinicians and scholars worldwide to be aware of as we strive to re-envision ACP, so that people are more comfortable engaging in ACP conversations. In terms of the content of tweets, we argue that there is a chasm between the biomedical and biopsychosocial elements of ACP, including patient narratives. If used properly, Twitter conversations and National Health Care Decision Day hashtags could be harnessed to serve as a connecting point among organizations, physicians, patients, and family members to lay the groundwork for the trajectory toward a *good death*.

## Introduction

### Background

Death and dying are unavoidable realities for all humans but remain taboo and subsequently avoided topics within most Western societies, especially in the United States, which has been referred to as a death-defying culture [1-4]. Prince-Paul and DiFranco [3] argued that death and dying are public health issues [3]. Indeed, complications with end-of-life (EOL) processes extend across the globe as there are social, economic, political, and historic perspectives influencing communication about mortality [4,5]. The stigma surrounding death, lack of awareness, inadequate health literacy, negative public perceptions, systemic barriers, and cultural differences also influence society’s reluctance to discuss issues related to potential EOL health care needs and expectations [5-14].

Since as early as the 1960s, many efforts have been made in the United States to promote engagement in advance care planning (ACP) conversations to encourage opportunities to express goals for EOL care and formally document those wishes in a written advance directive (AD) [4,12-14]. According to a (2017) consensus definition from a global panel of experts, “Advance care planning enables individuals to define goals and preferences for future medical treatment and care, to discuss these goals and preferences with family and health-care providers, and to record and review these preferences if appropriate” [13].

Part of this planning process includes the creation of an AD. According to the National Institute of Aging, ADs refer to written legal documents expressing one’s values and preferences related to EOL care, which can be modified throughout one’s lifetime [14]. This document is then retained and only goes into effect if a person is unable to speak for themselves. Recent efforts to promote ACP in the United States include *My Five Wishes, The Conversation Project*, *The Stanford Letter Project,* and *Death Cafes.* However, despite these efforts, low rates of ACP and the completion of ADs persist [15].

Presenting *opportunities* to engage in a public dialog about death and dying to normalize these difficult conversations and reduce the stigma surrounding death may improve public discourse and eventually lead to an improvement in ACP rates [3,16,17]. Specifically, recent work suggests that the presence of high engagement among health care participants throughout the EOL experience can increase the likelihood of a *good death* [17]. Although this does not mean that one’s death will be completely painless or without complications, it does help ensure that the dying experience is as good as possible for all parties involved. A particular opportunity to promote engagement in planning and preparing for EOL care in the United States is National Healthcare Decisions Day (NHDD), an observance dedicated to communicating about EOL by advocating for ACP [18].

Scholars have pointed to the potential of the internet, as well as social media platforms, to facilitate discussions, information sharing, and social support around challenging health topics, such as cancer [19-21], and reduce mental health stigma [22]. In particular, Twitter has been studied as a tool for communicating about cancer [20], human papillomavirus vaccines [23], and Alzheimer disease [24]. Recent work has indicated that Twitter can be a useful tool for health care participants to communicate about ACP, suggesting that “Twitter is a new avenue for patients, clinicians, and advocates to engage with each other to better understand each other’s perspectives related to ACP” [25]. Key to the diffusion of information and the creation of communities on Twitter is the use of hashtags, which could serve to unify discourse and induce a sense of support and belonging [26,27]. Coupling this with recent calls to reconsider approaches to ACP [9,28-30], we extend this work as this study situates Twitter as a social media tool and relevant NHDD hashtags as a mechanism to engage in conversations related to ACP.

### Achievement of a Good Death

Although possibly appearing contradictory in nature, the concept of a *good death* carries a great deal of significance for patients, loved ones, and health care participants alike [17,31]. Although each person’s perception may differ on how a good or successful death would be defined by them personally, there are commonalities with what this experience entails. Such experiences are constructed of elements related to physiological, social, existential, and spiritual components [32], which are then reflected in specific actions such as pain management, being in the presence of loved ones, and respecting the patient’s values and wishes [33,34]. However, a *good death* manifests itself differently for each person, considering their history, cultural background, attitudes, health conditions, and personal views and attitudes toward death [35]. Although much work has been dedicated to establishing a more positive death experience, the ability to die well continues to be inhibited by several challenges, including poor communication, physical and systemic barriers, lack of knowledge related to the disease trajectory, and discontinuity of care [17]. A recent commission put forth a report to help refocus on and find value in death. Five principles were offered to help re-envision death and dying, including “the social determinants of death, dying, and grieving are tackled; dying is understood to be a relational and spiritual process rather than simply a physiological event; networks of care lead support for people dying, caring, and grieving; conversations and stories about everyday death, dying, and grief become common; and death is recognized as having value.” [5]

In an attempt to make sense of the dying process and find value amidst death, we present the *Opportunity Model for Presence During the EOL Process* (OMP-EOLP) and argue that for a quality dying experience to take place, health care participants must be included at every step of a health care journey [17]. Opportunities for presence include integrating such conversations into the sociocultural context of individuals, which helps normalize and reduce the stigma of engaging in EOL conversations. Specifically, the biopsychosocial and spiritual elements of health care participants and their values and culture, including media exposure and language, make up the sociocultural context. As the conversation starts in a sociocultural context, it continues when either a terminal diagnosis is made or returns to a conversation regarding ACP throughout the normal aging process. Additional opportunities for presence during the EOL process include the place of care, knowledge about family members’ health status, and the moment of death. With high engagement at each step of the way and by having a *presence check*, the opportunity to die well is improved. We argue that from a public health perspective, ACP conversations using Twitter can influence engagement throughout the EOL process.

### About NHDD

Occurring annually on April 16 in the United States, NHDD exists to inspire, educate, and empower the public by providing information regarding the importance of ACP [18]. First founded in 2008, the NHDD was created with the goal of providing clear, concise, and consistent information related to health care decision-making [36]. Since then, the initiative’s goal has been to target the general public, health care providers, and facilities to provide free, simple, and uniform tools to guide this process [37]. Now managed by *The Conversation Project,* started by Ellen Goodman in 2010, NHDD is described as a *public engagement initiative* [18], which was started by Goodman when she and a group of colleagues started sharing personal stories related to *good* deaths or *hard deaths* they had witnessed. The NHDD serves to encourage patients to express their wishes regarding their health care. In turn, this effort also exhorts providers and facilities alike to respect such wishes, regardless of what is expressed or asked for. Although efforts related to NHDD comprise community interventions, interpersonal interactions, and in-person events, such efforts have also been conducted on the web. This includes the integration of web-based toolkits featuring ACP resources, as well as engaging viewers on social media platforms such as Twitter [25].

### Hashtag Activism and NHDD’s Presence on the Web

Key to the flow of information in web-based campaigns is the use of consistent and widely shared hashtags. From *#BlackLivesMatter* [26,27] to *#OccupyWallStreet* and *#MeToo* [38], hashtags have been used in recent years as an efficient way of solidifying networked activism and diffusing discourse around social issues. Beyond connecting social media users using similar linguistic symbols, hashtags are also useful for broadening discursive communities, for example, by attracting the attention of journalists and other elites [39]. Hashtags have been used to promote public health interventions, causes, and events and facilitate 2-way communication between public health officials and the public on multiple occasions, such as *#LiveFitNOLA* [40] and *#GetUsPPE* [41]. In fact, so potent is the use of hashtags for health campaigns and discussions that in the realm of vaccines, they were used both by those promoting science, such as the provaccine *#DoctorsSpeakUp* [42], and malicious actors who hoped to sow discord among Americans, such as the Russian Internet Research Agency’s use of the antivaccine hashtag *#VaccinateUS* [43]. Most recently, Cutshall et al [25] focused on ACP and brain tumor stakeholders with *#BTSM* (brain tumor social media), connecting such hashtag engagement and related activism efforts and extending it to advance efforts toward EOL care, specifically ACP.

## Methods

### This Study

This study examines the intersection of ACP Twitter conversations surrounding hashtags related to NHDD and how communicated tweets fit into the sociocultural context as an opportunity for engaging in EOL conversations. Specifically, it looks at how EOL, ACP, and NHDD are discussed among Twitter users and what conversations might be able to tell us in terms of individuals’ attitudes and potential behavioral intentions regarding ACP. In light of this, we pose the following research questions (RQs):

- RQ1: In regard to *NHDD*, what were the prominent topics of conversation in the tweets?
- RQ2: Who was leading the conversations regarding *NHDD*?

With this, we analyzed tweets published over a full year, between August 1, 2020, and July 30, 2021 (N=9713), to understand how social media had been used as a tool for promoting a day dedicated to making health care decisions, which we call discussions of ACP. For the purposes of this study, we use the term ACP to broadly encompass the whole process of engaging in conversations about EOL goals of care, regardless of whether an AD is completed.

### Data Collection

As our theoretical focus is on the unification of language around hashtags, we curated a list of hashtags that were centered on NHDD by reviewing what the holiday was about and which hashtags were used. We also went to Twitter to search for related terms and then collected all tweets containing the following hashtags: *#nhdd*, *#advancecareplan*, and *#goalsofcare*. To identify discourse around the NHDD that did not use hashtags, we also collected tweets using the exact terms *national healthcare decision day*, *advance care plan*, *goals of care*, and *goals-of-care*. Using these keywords, we collected all available tweets published between August 1, 2020, and July 30, 2021 (N=9713) on Twitter using the company’s academic application programming interface (7410/9713, 76.27% after removal of duplicates).

### Procedure and Measures

#### Identifying Themes in the Corpus

**Figure 1:**
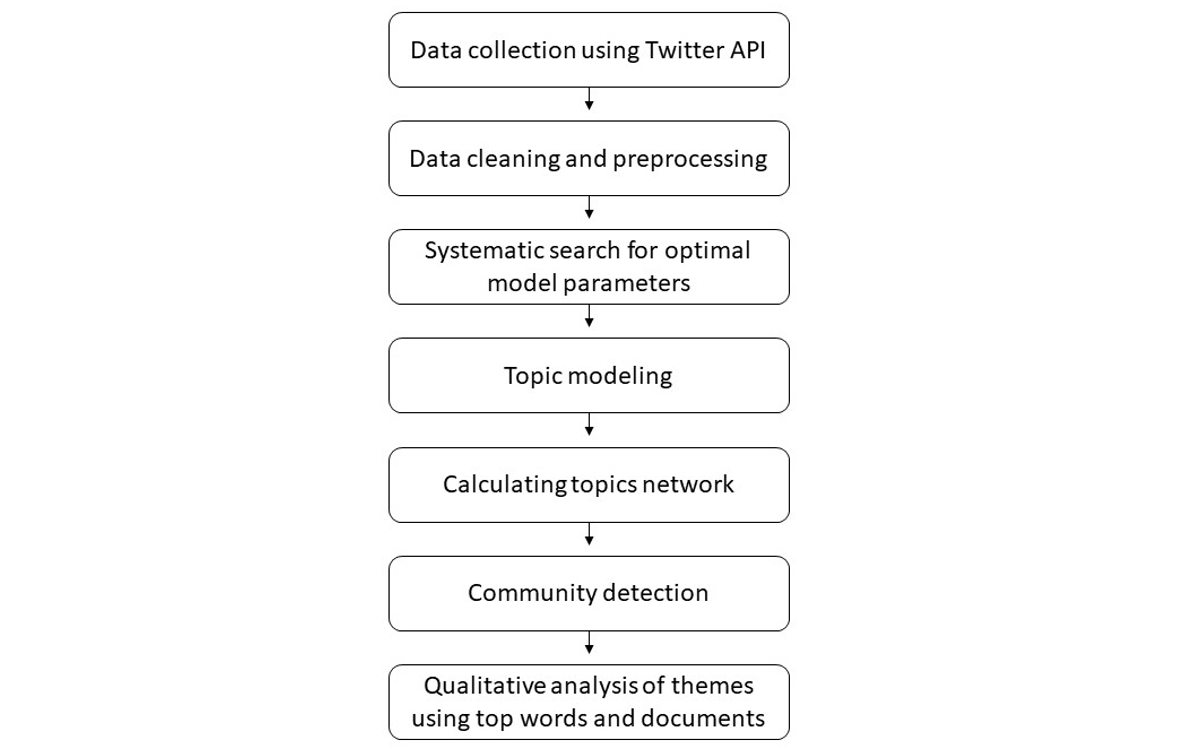
Overview of the Analysis of Topic Model Network approach and methodology. API: application programming interface.

Our inductive modeling of the corpus relied on the Analysis of Topic Model Network (ANTMN) framework [44]. The ANTMN method suggests a four-step process: topic modeling, topic networking, community detection, and qualitative analysis, as shown in Figure 1. Topic modeling (in this case, using latent Dirichlet allocation) is an unsupervised machine learning method that identifies themes in large textual data [45]. The modeling’s unsupervised, inductive nature makes it especially useful for work with corpora in which the set of possible topics and themes is not known a priori [46]. Topics are distribution lists of word probabilities based on their co-occurrences in the same documents (in our case, Twitter posts and tweets). Importantly, although the modeling stages were performed inductively and with no reliance on prior theory, after automated modeling, researchers interpreted the results by qualitatively studying the most common words related to each topic and the most representative documents for each topic. Harnessing the breadth of the data, we also looked at dynamic changes in prevalence topics and themes over time.

Following the standards in communication research [46], we preprocessed the corpus by removing stop words, punctuation, numbers, and symbols and converted all text to lowercase. We refrained from stemming or lemmatization based on the recommended best practices in topic modeling [47]. For hyperparameter tuning, aimed at accounting for the short nature of the texts examined [43], we used 5-fold cross-validation iterating over a range of topic numbers (from *k*=5 to *k*=100, in *skips* of 5) and various levels of the α hyperparameter (α=.01, .05, .1, .2, and .5). We found that the model with *k*=30 and α=.01 offered optimal results based on perplexity scores [43]. Topics were labeled qualitatively by examining the top words, unique words, and documents representative of each topic. To avoid biases in modeling, the topic model was conducted on a sample without duplicates (7410/9713, 76.27%), and analyses of changes over time were conducted on the full sample (N=9713).

In ANTMN’s second step, a network of topics was calculated based on the co-occurrence of topics in the same tweets (calculated as the cosine similarity over the document topic matrix). We used these similarities as links in the network where each topic served as a node and their co-occurrence as the link. To remove spurious links and reduce network density, we used a backbone method [48] to discard nonsignificant links. In the third step, we used a community detection algorithm based on the eigenvectors of matrices [49] to group the topics into broader themes. Finally, we qualitatively analyzed the model and its output and labeled each theme based on this close reading. Our qualitative analysis, described in the following section and in the *Results* section, is based on the automated identification of these themes.

#### Qualitative Analysis

Following the ANTMN approach’s fourth step [44], we engaged in qualitative discourse analysis [50], focusing on the language surrounding the making of health care decisions, the meaning surrounding ACP and EOL, and the context in which ACP conversations do or do not take place to understand the nuances of tweets related to NHDD in light of 3 specific theoretical and practical perspectives. The first is the NHDD mission and the ACP explaining the following:

National Healthcare Decisions Day [NHDD] exists to inspire, educate and empower the public and providers about the importance of advance care planning. NHDD is an initiative to encourage patients to express their wishes regarding healthcare and for providers and facilities to respect those wishes, whatever they may be [18].

Second, we focused on Twitter discourse in the conceptual framework of the OMP-OELP [17], specifically the sociocultural context of ACP conversations. Finally, we examined Twitter as a tool that can be used for activism, particularly in advocating for engagement in ACP. A total of 4 authors, 2 of whom were experts in EOL communication and 1 a PhD-prepared registered nurse with specialty certification in palliative and hospice care, reviewed the tweets and model to become familiar with the most representative texts and words associated with each topic. We were then able to begin to group similar ideas and content together. This process guided the labeling and definition of each topic. The final step in the qualitative analysis was reviewing the discourse for unique cases, defined as “data that demonstrate sharp contrasts with the major pattern that accounts for most of the data” [51,52], or, in this case, outstanding tweets that were relevant in the EOL process but were so exclusive that the nuance was important to take note of to see how the discourse remained thematically in the data set or stood on its own.

## Results

### Overview

Before analyzing the data in response to our RQs, we examined descriptive statistics indicating the most used hashtags, as well as the most liked and retweeted tweets, to better understand the discourse surrounding NHDD hashtags. Descriptive information of the gathered tweets is shown in Table 1.

**Table 1:** Descriptive information from the corpus.

Descriptive information	Value, n
Unique users	6974
Unique tweets	1986
Average character length	172

### Top Hashtags

We identified multiple shared hashtags related to different goals or aspects involved in or affecting ACP. The first top hashtag we saw was *#NHDD* (728/9713, 7.5%), specifically linking tweets to conversations about NHDD. The second was *#COVID19* (371/9713, 3.82%); the third was *#goalsofcare* (202/9713, 2.08%); the fourth was *#PalliativeCare* (160/9713, 1.65%); and finally, the fifth was *#NationalHealthcareDecisionsDay* (141/9713, 1.45%). It is important to note that there is some overlap in the hashtags used (ie, *#NHDD* vs *#NationalHealthcareDecisionsDay*) because of differences in capitalization, spelling, or abbreviations. The top 10 hashtags used in tweets can be found in Table 2.

**Table 2:** Frequency of the top 10 hashtags used in tweets from the corpus (N=9713).

Hashtag	Frequency, n (%)
*#NHDD*	728 (7.5)
*#COVID19*	371 (3.8)
*#goalsofcare*	202 (2.08)
*#PalliativeCare*	160 (1.65)
*#NationalHealthcareDecisionsDay*	141 (1.45)
*#AdvanceCarePlanning*	132 (1.36)
*#ICU*	128 (1.32)
*#advancecareplanning*	108 (1.11)
*#GOCCNJ*	106 (1.09)
*#hapc*	101 (1.04)

### Most Liked Tweets

We also analyzed different forms of engagement. This included tweets that received the most likes from other users across the data set. Shared here are the top 3 most liked tweets; the remaining tweets can be found in Table 3. First, the tweet with the greatest number of likes received a total of 893 likes and stated the following:

Maybe I’m crazy, but I think surgery interns should do a palliative medicine rotation. I learned how to have successful goals of care discussions from multi-disciplinary meetings where palliative was involved. It’s an invaluable skill that most of us don’t get formally taught.

The next most liked tweet was from another individual, which received 594 likes. Here, they stated the following:

You need to let them talk and listen carefully. After that, you should try to phrase the goals of care with your own words. “Based on what you told me, we should probably focus on comfort and make sure he doesn't suffer any more.”

Finally, the third most liked tweet was from the same user referenced above. This message received 589 likes and stated the following:

One of my favorite consults is goals of care BEFORE high-risk CT [cardio thoracic] surgery. Why palliative care/GOC [goals of care] before surgery? It is because we need to have a high-quality conversation, in very challenging cases. In my opinion, there must be 2 phases of conversations.

**Table 3:** Top 10 most liked tweets from the corpus.

Tweet	Likes, n
“Maybe I’m crazy, but I think surgery interns should do a palliative medicine rotation. I learned how to have successful goals of care discussions from multi-disciplinary meetings where palliative was involved. It’s an invaluable skill that most of us don’t get formally taught.”	893
“You need to let them talk and listen carefully. After that, you should try to phrase the goals of care with your own words. ‘Based on what you told me, we should probably focus on comfort and make sure he doesn't suffer any more’”	594
“One of my favorite consults is goals of care BEFORE high-risk CT surgery. Why palliative care/GOC before surgery? It is because we need to have a high-quality conversation, in very challenging cases. In my opinion, there must be 2 phases of conversations.”	589
“A fun little thing I like to do as a palliative consultant is base my recommendations on the patient’s goals of care.”	427
“Two most common questions I ask when accepting a patient to the ICU (from any setting): (1) did you address goals of care? (2) can you please turn off the maintenance fluids?”	398
“If you’re referring someone to me for a goals of care discussion I give you preemptive permission to discontinue their statin”	391
“A family meeting to determine goals of care over the PHONE (not even video) in a foreign language (with an interpreter) with an unknown number of people is a new kind of hell I hope to not revisit.”	347
“#PalliativeCare friends, if you're in the hospital and doing Advanced Care Planning, ask the patient if nursing students can come and listen. In my 4 yrs of nursing school I never learned how to talk about ACP or about goals of care. It's an invaluable lesson to learn. #hapc”	344
“Working in the ICU last month taught me a lot of things. Yes, all providers need more training in end of life discussions. But we also need to unlearn the ableism that influences our goals of care and quality of life discussions.”	342
“18. The number of families I updated today. 12. The number of days left on COVID unit 10. The number of minutes I sat down to eat. 6. The number of concurrent goals of care conversations I had in a 2hr span. 1. The one pt who told me she is ready to go. Time to rest for the day.”	275

### Most Retweets

Finally, another way we examined engagement was by evaluating the number of retweets in Table 2 among messages shared. Once again, we saw consistency in users, as the most retweeted tweets came from the same person with the second and third most liked tweets. As seen previously, this tweet received 127 retweets and stated the following:

One of my favorite consults is goals of care BEFORE high-risk CT [cardiothoracic] surgery. Why palliative care/GOC [goals-of-care] before surgery? It is because we need to have a high-quality conversation, in very challenging cases. In my opinion, there must be 2 phases of conversations.

The second most retweeted message received a total of 123 retweets and was from the *Journal for Geriatrics Clinical Science.* Here they shared the following:

Frailty is a key predictor of COVID-19 prognosis, and its assessment alongside measures of acute morbidity, rather than age alone, might help clinicians in offering realistic goals of care in hospitalized patients with #COVID19. #geriatrics.

Finally, the third most retweeted tweet was from *Intensive Care Medicine,* an international peer-reviewed medical journal for intensive care medicine. With a total of 119 retweets, they shared the following:

Long-term outcome of elderly [= 80y] #COVID19 pts admitted to #ICU: 6-month mortality...72% [likely underestimated] at upper end of recent literature on older critical pts. Data supporting more informed goals-of-care decisions for this #SARSCoV2 cohort

### Addressing the RQs

#### Overview

**Figure 2:**
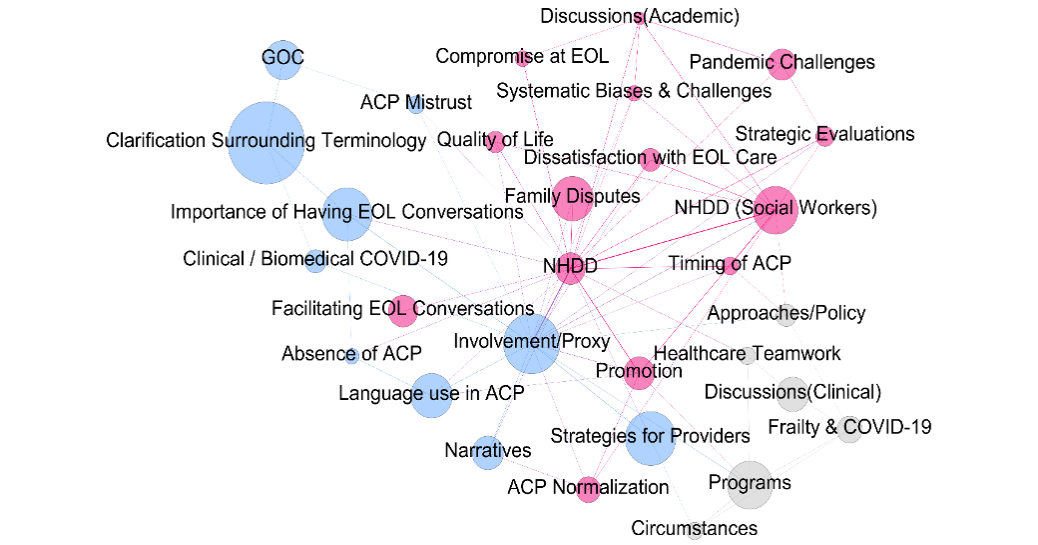
A topic network using the Analysis of Topic Model Network. The color of nodes indicates the community (theme) associated with each topic. Node size indicates topic prominence within the entire corpus. ACP: advance care planning; EOL: end of life; GOC: goals of care; NHDD: National Healthcare Decisions Day.

RQ1 asked what the prominent topics of conversation were in regard to NHDD during the studied year. A complete list of themes and their corresponding topics is provided in Multimedia Appendix 1. The ANTMN [44] model of 30 topics revealed 3 distinct themes. On the basis of a qualitative analysis of the most representative texts and words, we were able to label each of the 30 topics. Then, upon further qualitative analysis of the 3 themes, we were able to appropriately label and define 3 overarching main themes in the network to encompass conversations. The overarching themes (Figure 2) were *importance and promotion* (red), *the surrounding language* (blue), and *systemic issues* (gray). Each of these is described in greater detail in the following sections.

#### Importance and Promotion

The most prominent overarching theme within the corpus was *importance and promotion* (red). Such conversations centered on the notion of NHDD itself, promoting the day among users and highlighting various elements of the ACP process. In addition, in this theme, conversations iterated why ACP is a necessary component of health care interactions, why it should be implemented and not simply limited to the EOL process, and challenges that thwart ACP efforts and success rates.

For example, many users shared information about NHDD and its purpose, such as the following tweets:

Today is #NationalHealthcareDecisionsDay. This recognition is designed to educate and empower the public about the importance of ACP. For more information about National Healthcare Decision Day and how to start planning....

Advance care planning can reduce a family’s anxiety, depression, and stress“. #PCC4U

Module 4 | Activity 6: Advance care planning and goals of care....

As shown previously, these conversations aim to increase the audience’s awareness that NHDD was taking place; what it was; why it was promoting ACP; and subsequently, why ACP is such a vital component of the health care process.

Conversations using this theme also introduced the idea of creating an AD, including the timing, normalization, and facilitation of and compromises and challenges of EOL:

Getting advanced directives is complicated and often generates disagreements w/in family in terms of who has power of attorney. Important to have these in place including goals of care and communicate well.

The nodes with the highest frequency included NHDD (social workers), family disputes, NHDD, promotion, and facilitating EOL conversations. On the basis of the model, we see that the relationships among NHDD, NHDD (social workers), and promotion is strong. The intersection of the timing of ACP between NHDD (social workers) and promotion illustrates that timing is an important part of the planning process.

The results of the discourse analysis revealed that the promotion efforts using NHDD hashtags reflected the mission of NHDD, which is to educate, inspire, and empower people to have ACP conversations. What we also see is the identification of problem areas or issues with ACP, which can be helpful to those initiating conversations from a clinical perspective and for awareness of the current state of ACP; that is, an agreement that having conversations is important but not that simple. Having one or more conversations about EOL wishes does not guarantee a smooth or high-quality EOL process. This is critical when applied to the OMP-EOLP, and public dialog is related to EOL taking place [3,14,16], in this case, via Twitter. The opportunity for presence is achieved through social media engagement. However, this is only 1 factor in the OMP-EOLP, and the continued high level of engagement of health care participants needs to follow the EOL trajectory. Although we cannot evaluate the success of this in this study, we argue for both academics and clinicians that an understanding of conversation at a public health level is necessary so that future efforts can be tailored more effectively to meet the needs of health care participants. A balance between promoting the positive aspects of ACP and understanding the barriers to ACP, including the ongoing pandemic, is essential.

#### Surrounding Language

The second theme, *surrounding language* (blue)*,* focused on the language used for clarification and guidance within ACP conversations. Specifically, we examined the linguistic choices and phrasing used when communicating about ACP. Within tweets using these topics, a large area of discussion described the need for clarification surrounding terminology. For example, dialog encompassed distinguishing goals of care from *code status* (physician orders for resuscitation or *do not resuscitate* [DNR]) and the continuing need to define goals of care for patients or care residents. Even among professionals, conversations noted how there were misunderstandings about what ACP entailed and how it differed from other aspects of care, particularly within the context of the patient’s diagnosis, prognosis, treatment options, and care needs. For example, a user tweeted the following:

Palliative care in R/R [relapsed/refractory] aggressive lymphomas:

- PC ≠ Hospice care or end-of-life care

- PC integration is low: too late and less than in solid tumors

- Identify triggers for goals of care discussions

- Collaboration between specialists.

Although most of this dialog pertained to clinical conversations (ie, strategies for providers, clinical and biomedical conversations related to ACP during COVID-19, and involvement of health care proxies), there was a small portion of dialog reflecting on patients’ experiences with ACP. We found that ACP was discussed in light of personal narratives, noting their experiences and stories of how the presence or absence of ACP affected the dying experience. For example, a user tweeted the following:

From a darling, deteriorating, elder contemplating goals of care “Tell me, sweet nurse, shall I die slowly or quickly?” I choked back my tears and replied “How about a goal of peacefully and let time do what it will.” Then we just sat in silence and held hands.

Many narratives, such as the abovementioned one, describe sad, emotional experiences when the patient is either close to death and is forced to consider goals of care and EOL wishes or when a practitioner or loved one reflects on poor death experiences that could have been made better by successful ACP. Shared publicly, these narratives could act as a unique educational tool, harnessing storytelling to emphasize the benefits of ACP, as well as the struggles associated with completing it at later stages. Other narrative efforts included video games or apps developed to make ACP conversations easier, adapting more of an entertainment–education approach to further ACP efforts, which communicates the importance of ACP in a different language rather than in clinical conversations.

The results illustrate another way in which language clarified that the realities of ACP were through expressing mistrust in ACP processes, claiming that if they (patients) filled out ADs, it was an initiative for physicians to reduce their level and quality of care. This is an alarming misperception that emerged from the data. Furthermore, this mistrust appeared to be fueled by the misbelief that engaging in ACP would expedite the dying process for the patient. Such tweets featured charged and accusatory language, which points to misinformation surrounding ACP and EOL processes and provides insights into the potential reception of such information. An example included a health care professional tweeting the following:

Sometimes people mistakenly think DNR means “don’t do anything.” Remember, DNR only goes into effect when a patient codes [cardiac arrest]. A DNR order is not a substitute for a goals of care discussion. They are not the same thing. #MedTwitter #CriticalCare #GoalsOfCare

This tweet was part of a larger conversation, discussing the misunderstandings of concepts such as a DNR order and how having a DNR order does not mean that physicians will not work as hard to care for them if completed. Identifying such misunderstandings in ACP demonstrates how Twitter can be used as a tool for activism to clarify the purpose, definitions, and information for those in the community.

Our results indicate that the language surrounding ACP tended to portray it as important but not without challenges, as evidenced by the fact that NHDD is a term that is euphemistic in its own right. By avoiding the use of clear terms surrounding death and dying, although well-intentioned, the lack of clarity can perpetuate confusion among health care providers and the public alike. On the basis of qualitative analysis, goals of care were intertwined with ACP, and a point of discussion and disagreement is whether goals of care equate to ACP. Other similar tweets shed light on how various social issues, such as racism and prior negative health care experiences, also fuel this mistrust, which leads to greater barriers that get in the way of the ACP process. The connection with the OMP-EOLP is that the sociocultural factors of health care participants are at play in ACP conversations on Twitter. It is critical for health care providers to be aware of this mistrust in underrepresented communities. For example, the well-documented mistrust of the health care system in the African-American community stems from unethical research practices such as the Tuskegee Syphilis study [53]. Support for this connection is illustrated in the Social Determinants of Health framework, wherein there are factors at play that are not biomedical but instead, a result of where we live, work, and age that influences our health [5,54-56].

#### Systemic Issues

The third overarching theme closely aligned with language use was *systemic issues* (gray). In examining these conversations, such issues or barriers related to ACP were discussed within clinical settings. The conversations documented issues related to the settings in which care was taking place, disparities in care with regard to socioeconomic groups, and subsequent costs related to care. For example, a tweet explained how “many barriers exist [in ACP] due to implicit bias. Lack of appropriate testing, follow-up, and assumptions about goals-of-care – all leading to poor outcomes....” In other words, owing to perhaps prior negative health care experiences or negative attitudes toward EOL discussions and planning, patients can subsequently experience poor outcomes at the EOL. Issues related to COVID-19 and frailty were also common, which elaborated on care options and decisions related to patients diagnosed with COVID-19. Current research related to ACP and COVID-19 indicates that the number of people filling out ACPs is on the rise; however, that does not equate to a successful EOL experience [57].

On the basis of the qualitative results, we see systemic issues uniquely related to marginalized populations, including prisons, specifically, how these populations faced greater barriers in health care settings, which were exemplified at the EOL [58,59]. Further complicating access to EOL care in prisons is COVID-19, when, for the first time, most prisoners are aged ≥55 years, which also begins to overlap with the most vulnerable population for COVID-19 [60]. For a population that includes those who are most vulnerable to COVID-19, with difficulties in practicing social distancing in addition to chronic illness and access to health care, the barriers to ACP are overwhelming.

Another unique consideration for ACP is in the context of Alzheimer disease and dementia-related care. In our data set of the 30 most representative tweets on each of the 30 topics (900/7410, 12.15%), Alzheimer disease was the only specific disease called out, aside from COVID-19, when discussing ACP. However, only a small number of tweets in our manually coded corpus mentioned this (5/7410, 0.07%). As Alzheimer is a terminal disease that attacks the brain and memory functions, we argue that the need for ACP is much stronger. Previous studies on Twitter and Alzheimer disease indicate that there is a stigma associated with the disease [61]. Topics in this theme called attention to barriers in conversations, as well as the differences in care that emerged. Future work should consider diverse and marginalized populations, their unique needs, and how intervention and communication materials may need to be targeted and/or tailored to make processes, such as ACP, more accessible and achievable. In addition, owing to negative past experiences or distrust in health care–related entities, special precautions and care may be needed to facilitate conversations.

### Leading NHDD Conversations

Next, RQ2 asked who was leading these conversations regarding the NHDD. Overall, the users appeared to be mostly health care professionals and health-related organizations. However, only 4.09% (303/7410) of tweets were from verified accounts, wherein the identity was verified from Twitter as a public figure or institution, and the remaining 95.91% (7107/7410) were from unverified users; that is, users promoted NHDD on their own as an individual Twitter user, and the message did not directly come from a public figure or institution. From an activism standpoint, NHDD appears to be common among health care professionals but is not promoted specifically by verified accounts. Although this study cannot draw definitive conclusions about the individuals who were tweeting, the heavy use of medical jargon and references to personal experiences working as clinicians suggest that many users were health care professionals.

From the analysis of tweets, we observed dialog among what appeared to be clinical health care professionals, presumably physicians, discussing the medical treatment plans of patients. We also observed a back and forth in tweets wherein messages were congratulatory in nature or provided shoutouts to other professionals on academic-related achievements (ie, conference presentations and paper publication). Tweets were saturated with heavy medical jargon and abbreviations such as *CT,* which means cardiothoracic; *#pallonc*, which means palliative oncology; *#hapc*, which means hospice and palliative care; and the following example, which is full of medical jargon and is further complicated by shorthand for words and potential typographical errors:

Keep sats 90 or higher, dex 6mg for 10d if o2 < 92-94%, early GOC talk, chemical dvt ppx for all, remdes maybe?, if really considering abx check procal (only ~2% have bacterial CAP), keep net neg, trial proning, HFNC?> NIV, more goals of care :(

Loosely translated, the above passage is referring to treatment for a COVID-19 patient and reads as follows:

It is important to keep oxygen saturation above 90%, to administer dexamethasone (which is a steroid) 6 mg for 10 days if oxygen saturation and lt (unclear, could be goal for oxygen saturation level) 92-94%, early goals of care talk, chemical deep venous thrombosis prophylaxis for all, remdesivir maybe? If really considering antibiotics check procalcitonin (only around 2% have bacterial community acquired pneumonia), keep net negative, trial proning (lying face down), high flow nasal cannula & gt (unclear, could be referring to gastrointestinal tube (gt) but it is unclear, could also be typo for gtt (drops)), non-invasive ventilation, more goals of care. :(

The examples illustrate how such conversations among physicians, scientists, and those with occupations related to the medical field are a part of the conversation, but those without the lexicon are unable to follow and understand the conversation.

On the basis of individuals identifying themselves as social workers in their tweets or tweets from large social work organizations, another profession that appeared to be leading conversations regarding ACP and NHDD was that of social workers. Research indicates a lack of consensus among health care practitioners as to who is best suited to lead ACP conversations [62]; however, we see that social workers are currently a part of this process, which past work looking at ACP may have overlooked. Here, social workers may have a large presence in tweets as their profession generally involves them in EOL conversations and, subsequently, in ACP and the completion of AD forms. An example of the presence of social workers included a tweet from the National Association of Social Workers’ Colorado chapter, which stated the following:

April 16th is National Healthcare Decisions Day, which #NASW supports. #NHDD

inspires, educates empowers the public and providers about the importance of advance care planning. #Socialworkers have an important role in advance care planning

#Covid19.

By identifying who is leading conversations for NHDD, we can identify a gap in the united front of health care professionals and laypersons advocating for EOL conversations while also educating the public about EOL resources. Knowing who is involved in such conversations is important as it allows us to better target resources for pre-existing conversations related to EOL wishes and planning. Combining efforts on the part of social workers and other medical practitioners may strengthen and increase the chances of not only AD completion but also following through on adhering to wishes that patients have put forth. We observed a lack of connection in the use of hashtags between clinical (biomedicine) and psychosocial–spiritual providers and family members, perpetuating silos separating the approach and process of ACP.

**Figure 3:**
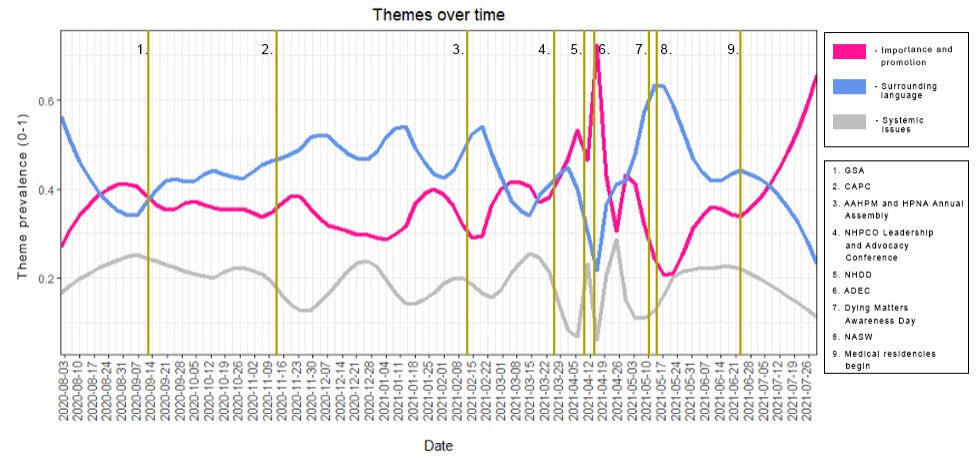
Frequency of themes measured over time from August 2020 to July 2021. Yellow lines indicate professional and academic events coinciding with the timeline. AAHPM: American Academy of Hospice and Palliative Medicine; ADEC: Association for Death Education and Counseling; CAPC: Center to Advance Palliative Care; GSA: Gerontological Society of America; HPNA: Hospice and Palliative Nurses Association; NASW: National Association of Social Workers; NHDD: National Healthcare Decisions Day; NHPCO: National Hospice and Palliative Care Organization.

To examine changes in the composition of tweets over time in terms of thematic use, we examined the frequency and thematic content of tweets throughout the 1-year period and overlaid the major conferences related to NHDD and EOL care (Figure 3). On the basis of this, we can see the spike in tweets around NHDD itself and other conferences taking place throughout the year (indicated in yellow in Figure 3). The problem is that the movement in tweets was not consistent, and presence was more common around professional and academic events than appearing to be a normal or consistent part of the dialog between professionals and laypersons alike, further supporting the notion that ACPs are a siloed effort.

## Discussion

### Comparison With Previous Work

NHDD is a niche hashtag that promotes a day in the United States dedicated to educating and empowering people to consider their options for making health care decisions in the event that they are faced with a terminal diagnosis or traumatic event that leaves them unable to speak for themselves. We argue that ACP conversations using Twitter, from a public health perspective, can influence engagement throughout the EOL process. On the basis of the results of the mixed methods study, we argue that who is sharing the information and *how* they do so are important in the engagement process.

The analysis indicated that most tweets were from health care organizations and appeared to be from medical professionals. However, we cannot definitively make assumptions about each user’s profession based on the corpus. That said, based on the content in conversations and seeing as it was medically based and filled with technical jargon, we can determine involvement as some form of health care provider or practitioner. In addition, individual users sometimes referenced their own experiences on the job either in hospitals or medical settings, such as medical students or acting physicians. The involvement of such professions in these conversations would make sense, given that Twitter is a social media platform that is used for engaging in public, large-scale, health-related, and oftentimes stigmatized conversations (Tenzek et al, unpublished data, 2022) [19,61]. In practice, this can be helpful for health professionals who need to locate continuing education credits or are interested in attending workshops related to ACP. Furthermore, the social work profession had a large presence in the analysis. Scholars have argued that any health professional could initiate a conversation related to ACP, which is critical in light of COVID-19 [63]. Research suggests that any one of the team members may be the one to engage in a conversation related to ACP with patients, and recently, chaplains have been identified as key professionals in helping with ACP, moving ACP conversations upstream [3,5,57]. We argue that being aware of the opportunities for engagement at different times in the health care experience becomes important for a quality EOL, and collaboratively communicating within the team becomes even more critical to providing quality care [17].

### Language, Barriers, and Activism

Next, *how* the message is communicated is also of great importance. Recent work suggests that language choice, including specific words and simplicity, is critical to how people receive messages related to ACP [64,65]. The results of this study point to the presence of information about this incredibly difficult, complex, and stigmatized topic, which is shared in ≤280 characters. We see in the current results is that it is mostly professionals and clinical terminology about the EOL process from health care providers’ perspectives, generally independent from overarching health care facilities or organizations. The professionals who had the right jargon could follow and contribute to the conversation; however, those without the medical dictionary and appropriate hashtag legend to make sense of all the information in tweets may not receive the messages or bypass them if they cannot understand. This means that individual physicians and health care workers are taking this initiative upon themselves to share with and educate others; however, this may be problematic. This study contributes to previous research that argues that engaging in public dialog about ACP must meet the public’s perceptions and beliefs about ACP and not of those who already believe ACP is important and have taken action [7]. Furthermore, upon observation, the NHDD hashtag does not directly communicate to audiences that it is an effort to help people plan for death. We should question whether the language choice was intentional in an effort to help audiences be more receptive to a goal-related conversation about health care decisions in general or if the avoidance further reinforces the taboo nature wherein US culture and societies silence this difficult conversation (Tenzek et al, unpublished data, 2022). On a global scale, we see efforts in England called *Dying Matters* and *Good Grief* and *Good Death* in Scotland, where there is no question about what will be discussed. We argue that in the sociocultural context, using language as an opportunity for engagement is critical, and further studies should examine people’s perceptions of willingness to talk about dying based on the hashtag being used.

Although the hashtag NHDD promotes ACP conversations, what we also see is the identification of systemic barriers, including sociocultural elements related to literacy levels, misinformation, policies (including Medicare reimbursements), and trust among health care professionals. Our analysis revealed unique ACP considerations, including patients with Alzheimer disease and prison populations, which are complex and stigmatized issues in health care. Using the Social Determinants of Health framework [5,54-56] in connection with the OMP-EOLP, we suggest a shift in ACP conversation to focus more broadly on the issues of literacy, access, and trust in health care as a resource for individuals. A recent study found that although 80% to 90% of people may be aware of ACP and believe that it is important, less than half (10%-41%) actually named a proxy or filled out a form [7]. It appears that education and awareness may not be the biggest barriers to ACP; people are aware that it exists, but there may be little action toward identifying specific wishes for care along the EOL continuum from diagnosis through death [17]. Morrison et al [30] argued that unless the health care system supports goal-centered care and provides resources to follow through with physician-patient conversations and consequential delivery of care, ACP outcomes will fall short of the intended goal; that is, providing the care at EOL that the patient wants. We see this conversation taking place on a global scale, as internationally, there are efforts to bring value back into the EOL [5].

Finally, in terms of activism and based on the argument of having to address ACP at the public health level, Grant et al [7] suggest, “public messaging that introduces these services to the public should differ from the skilled communication that clinicians perform at the bedside of patients with a serious illness” [7]. In doing so, part of the health care experience is ACP throughout a lifetime, not only at EOL, thus shifting ACP conversations upstream [3,66]. This is incredibly important for clinicians and scholars to be aware of as we strive to re-envision ACP so that people are more comfortable engaging in ACP conversations. In terms of the content of tweets, we argue that there is a chasm between the biomedical and biopsychosocial elements of ACP, including patient narratives. Aligning with Cutshall et al [25], if used properly, Twitter conversations and certain hashtags can be harnessed to serve as a connecting point among organizations, physicians, patients, and family members. It is a difficult but worthy effort to lay the groundwork for the trajectory toward a *good death*.

### Limitations

Although this study served as an analysis to examine conversations surrounding NHDD, this work explored a snapshot in the time surrounding NHDD in 2021. However, NHDD has been occurring for 13 years. The analysis also captured NHDD hashtags during the ongoing global pandemic, when death was omnipresent across news platforms [5]. We were not able to compare hashtag use in a pandemic, nor were we able to definitively determine the authors of the tweets. On the basis of our findings and a recent report of the *Lancet* Commission to reimagine death, the pandemic may have further stigmatized, instilled fear, or created hesitancy toward engaging in EOL conversations [5]. Future work should explore prior conversations to reveal trends or changes in conversations over time and delve further into the implications that COVID-19 has had on how we discuss dying. Our observation is that the tension between recent efforts at reimagining and upstreaming ACP, both in the United States and on a global level, and the negative impacts of COVID-19–related deaths present new challenges in finding the value in dying.

A second limitation is that the tweets examined were only from publicly shared accounts. This means that other conversations about NHDD and ACP could exist that we could not capture. This may explain why most tweets were from professionals or organizations where the day was promoted and known within the context of EOL. The key is figuring out how to continue to promote and inform the public in the United States about NHDD and, subsequently, about ACP. As the use of social media continues to be part of our daily lives, we argue that Twitter can also be used to promote EOL processes. Third, although Twitter is becoming more popular as a way of engaging in discussions encompassing diverse perspectives without geographical boundaries, current trends in social media use indicate that other social media platforms such as Facebook and Instagram have more users. In addition, with the rise of TikTok during the COVID-19 pandemic [67], researchers should consider moving toward building a bridge that connects social media messages related to ACP across platforms and users alike. Finally, future studies may go beyond the text of tweets to examine external links [68], visual communication [69], and communication across different social media [70].

### Conclusions

Overall, this study explored Twitter conversations surrounding the NHDD and, subsequently, ACP. Twitter conversations were centered on topics related to promotion, language, and systemic issues. Tweets portrayed ACP as a crucial and necessary tool, especially within the EOL context; however, Twitter users also raised concerns and criticisms about ACP that require additional research to disentangle and create specialized campaigns to address barriers and bias in the health care system. Although several prominent conversations did occur across Twitter, the present analysis shows that there is still work to be done regarding the successful integration of ACP into common everyday practice and conversation. This study allows us to see what is present and subsequently lacking in ACP conversations. In turn, the findings can aid in structuring interventions to help better promote NHDD and help practitioners and patients alike reap the benefits of ACP. Ultimately, the findings can provide more opportunities for conversations about ACP and hopefully encourage open communication about EOL decision-making and planning as a foundation for future presence through the EOL process.

## Appendix

Multimedia Appendix 1 Overarching themes, individual topics, theme notes, and exemplar tweets from each topic.

## Abbreviations

ACP: advance care planning
AD: advance directive
ANTMN: Analysis of Topic Model Network
DNR: do not resuscitate
EOL: end-of-life
NHDD: National Healthcare Decisions Day
OMP-EOLP: Opportunity Model for Presence During the End-of-Life Process
RQ: research question
