# Supplementary material for: Exploring Web-Based Twitter Conversations Surrounding National Healthcare Decisions Day and Advance Care Planning From a Sociocultural Perspective: Computational Mixed Methods Analysis
Source: JMIR Form Res. 2022 Apr 13;6(4):e35795. doi: 10.2196/35795 (PMC9047726; doi:10.2196/35795)
Supplement: Multimedia Appendix 1 [file formative_v6i4e35795_app1.docx]

**Appendix A***Table depicting overarching themes, individual topics, theme notes, and exemplar tweets from each topic*

| Overarching Theme | Individual Topic | Theme Notes |
| --- | --- | --- |
| Importance  & Promotion | NHDD | Encouraging ACP; day recognition; celebration; observance |
|  | 1. Decide. Write. Share. These steps can reduce the stress and burden during end-of-life by considering the medical care your loved one might want when facing a medical emergency. Learn more about the importance of #acp here: <https://t.co/xEs0U1gvVj> #eolcare #nhdd #hapc #hpm <https://t.co/o8O0a1rNEw> 2. Planning ahead of a health crisis = WIN! It's never too late to make decisions regarding your health, in case you aren't able to make them in the future. This gives you and your family peace of mind, nothing beats that! Make the right decision today! #health #NHDD <https://t.co/qaFYTAgLJs> 3. National Healthcare Decision Day, or NHDD, an initiative to encourage people to express their wishes regarding healthcare and for providers and facilities to respect those wishes, whatever they may be. Visit https://t.co/mTEPgrKcYf to learn more! #NHDD2021 #HPM #AdvanceDirectives https://t.co/IVzCQwedkZ | |
|  | Promotion | Encouraging ACP; ACP  planning & promotion |
|  | 1. "Advance care planning can reduce a family's anxiety, depression and stress". #PCC4U Module 4 \| Activity 6: Advance care planning and goals of care <https://t.co/XeOEFROE9q> #palliativecare #education #acpweek21 @ACPAustralia #CPC @ QUT <https://t.co/V5VaPLBxJ9> 2. We encourage them to share Advance Decisions and Advance Statements with clinicians (obviously) and they can be stored on NHS computers. Videos can't - and mostly the documents they take to clinicians mean they have direct convos with those people about goals of care. 3. Today is #NationalHealthcareDecisionsDay. This recognition is designed to educate and empower the public about the importance of advanced care planning. For more information about National Healthcare Decision Day and how to start planning, please visit https://t.co/R5dI1Pu5Fl. <https://t.co/TEXSkBVwbl> | |
|  | Systemic Biases & Challenges | Needs in care; minority challenges; systematic bias |
|  | 1. Is Implicit Bias Contributing to Time Disparities in Goals-of-Care Conversations With Minority Patients? Read a conversation with Dr. Cardinale B. Smith, a guest presenter in our upcoming AAPCCC webinar on #implicitbias: <https://t.co/vGKMJzWkAd> 2. On this #NationalHealthcareDecisionsDay, remember this one thing. You don't have to go where you're told to go. You have the right to choose the healthcare provider you feel is best to resolve your specific issue. #healthcare #NHDD #physicaltherapy #decision <https://t.co/GRDLZSnkcz> 3. The #healthcare system can be difficult to navigate. We're here to help. Check out our glossary of commonly used words and phrases when making an #advancecareplan. <https://t.co/qqTeQuWZoS> | |
|  | Strategic Evaluations | Careful evaluations of need for GOC convos; EOL care, GOC, patients |
|  | 1. MomRobe I used to ruminate about the ex-23-wk, in-utero drug-exposed apprehended kiddo who would arrest multiple times a shift, but no goals of care in place. When you've gotta be snowed and roc'd to be alive... I often wonder if they're still coding the kid every shift.G4 2. Prolonged Unconsciousness Following Severe COVID-19: After cessation of sedation, 6 patients recovered consciousness 8 to 31 days later - suggests need for caution when prognosticating and discussing goals of care with families. #curingcoma #COVID19 <https://t.co/a9s2QMcP6M> 3. A compelling essay by @guptaarjun90 , highlighting the value of teamwork, negotiating the goals of care and managing symptoms for people with serious illness, and the humbling nature of medicine (and life)."But plans, it turns out, are only plans." #pallonc <https://t.co/m3Apc9bDDW> | |
|  | ACP Normalization | Facilitating conversations; not waiting until death; normalizing; promoting organizations/events to help with this |
|  | 1. #NHDD reminds us advance care planning is something all families should discuss, regardless of whether you’re facing a terminal illness or not. Ask your primary care doctor about advance care planning or call 203-852-2764 for assistance from a #NorwalkHospital case manager. <https://t.co/t1eiE42QPh> 2. Tune in TOMORROW at Noon ET for HFA's live webinar, Spirituality as a Foundation for Advance Care Planning. Learn more about this live CE program and register here: <https://t.co/XjkHjL8jUa> @PastorCorey @JessicaZitter @KnowYourWishes #acp #eol #hapc #advancecareplanning #nhdd <https://t.co/d15pkQo3pV> 3. Are you part of a community group looking to help underserved communities with advance care planning? Check out the eligibility to host a Project Talk Trial event here: https://t.co/ylZZkzflpK #hapc #eol #advancecareplanning #nhdd #acp <https://t.co/8d6ltGEk09> | |
|  | Dissatisfaction with EOL Care | Role of prognosis in ACP; senior care; homecare; GOC; aging and homes; mistrust, misunderstanding, misinformation (negative connotations) |
|  | 1. Time spent with pts best used educating about disease &; Rx options, goals of care, etc; but if you must share info about how NP care is ‘equal if not better’ than medical doctors, I find visual cues always impactful, helps with retention. <https://t.co/9AtNpRLtml> <https://t.co/UHwgzczt2p> 2. Many people don’t understand that many seniors in LTC homes have Goals of Care designations. Those listed as C1 or C2 will be given no cure if they catch flu, Covid or if they have stroke, heart attack, Cancer, liver disease. Only comfort is offered. <https://t.co/PCPryUR9yr> 3. Unique risks of aging: immune senesce, congregate living, chronic health conditions &amp; invasive devices (exposure to health care setting), limited physiologic reserve. Goals of care &amp; potential for meaningful recovery should guide overall approach. | |
|  | Discussions (Academic) | Professional discussions; academic and clinical professionals talking; research; reports; researchers |
|  | 1. Deprescribing=essential component of good prescribing to reconcile medication use w/goals of care in older adults. Observational studies of admin. healthcare data uniquely positioned to address risks &amp; benefits knowledge gaps of continuing vs stopping medications. #PittHSRseminar <https://t.co/HonduFQqlF> 2. Shout out to @amy_linsky for making collaborative grant writing downright fun! Let’s hope our @vahsrd proposal to evaluate impact of #covid on goals of care conversations follows in footsteps of successful grants from @VA_CHOIR MPI powerhouse duo @hjmull &amp; @BranchWestyn! 3. Thank you @capitalhealthnj for hosting @BarileMd, #GOCCNJ's Founder/Chief Medical Officer, where he presented "POLST 2.0: Applying a Four Step Model to Improve POLST Form Completion" during grand rounds. #goalsofcare <https://t.co/VbnYPLWEGS> | |
|  | Quality of Life | Importance of considering QOL; clinical speak – stroke; working with patients; provider support; struggles |
|  | 1. I don’t think you can, however AS is certainly not helping. If he is supported well, oxygenating, perfusing, lactate is clearing, swan indices are improving- certainly could monitor. However, if deteriorating, may be few options. Salvage BAV/TAVR Vs goals of care discussion. 2. If anything, I would think interdisciplinary rounds in the midst of a pandemic of a novel virus would enhance safety, keep the team focused on the goals of care, enhance comms with family, cross check each other to reduce errors, etc.? 3. #EAACI20 Patients may well have different expectations for the benefit of a biologic therapy. We as caregivers are looking to reduce exacerbations. They, as patients, may be hoping to give up using inhalers. Communication of goals of care is key. #SharedDecisionMaking | |
|  | Compromise at EOL | Tradeoffs; physicians critiquing prescriptions & ACP; |
|  | 1. i never know who will understand and who will give up on helping me bc i don't treat their word as gospel. i think an ND doctor would be much more understanding and hear me out when i explain my needs and goals of care. its always such a gamble, its awful and scary 2. I do seem to see a small generation of young adults who were aggressively medicated for more situationally mediated issues and have a range of issues and limited prospects related to that. that comes down to the goals of care not meds in general 3. Having to confer on ‘Goals of Care’ via texts is not easy on anyone. Fortunately (we) are in agreement and things are being done! Never, ever, take the people you love for granted. Love my (baby!) @FavSisYeg sister, Hubby#1, our Dad and our combined families. Thank U #yeg peeps. | |
|  | Timing of ACP | When to discuss GOC; advocacy; promotion; tools; age; elderly care; respect & planning with older adults |
|  | 1. Depends on pt goals of care. I think for healthy pts am inclined toward debulking if you can render NED and then pick a time for "adjuvant" imatinib. But could easily make opposite case esp if NED by PET 2. COVID-19's impact? Nearly 1 in 3 Americans say they’re now more likely to discuss &amp; document their wishes &amp; values for end-of-life care. Find more from our survey. Our experts are available to facilitate difficult conversations. #VITAS #NHDD #TimeToTalk 3. First theory- Previously, a large percentage of hospitalizations were 70+: appropriate goals of care meant no ICU admission. Now vaccinated. 2nd theory: admissions bypassing wards and straight to icu?? 4. Fewer than half of all people in the USA have a documented #advancecareplan. Hospitalization offers an opportunity for physicians to initiate #ACP conversations. And now there's a theory-based adventure video game to help &gt;&gt; https://t.co/Wu0yi2fVJq <https://t.co/r7Nyav4owa> | |
|  | Pandemic Challenges | Discussions about how difficult to do GOC in pandemic; clinical back and forth – communication; clinical dealings and conversations regarding treatment |
|  | 1. Frameworks unifying clinical data allow revisitation of attribution and prognostication. Is nausea from chemo or brain mets? Pericardial effusion as progression of malignancy or a toxicity of therapy? Changes management and goals of care dramatically in my experience. 2. Looks like GB cancer with intrahepatic mets and likely duodenal invasion with GOO. You aren’t going to help this poor patient with a complex major liver resection, etc. Wouldn’t do a central anyway as it looks like a met in seg 6. Biopsy, NGS, and goals of care discussion. 3. In fact. I have never in my career had a “goals of care discussion” with a family. In an unclear situation like TBI specifically, I introduce my roll as an extra layer of support and to help with communication across teams. | |
|  | Family Disputes | Family issues and providers who should discuss ACP/GOC; arguing; bickering; misunderstanding; patient wishes |
|  | 1. @bereevapp Group discussion with family on general wishes and goals of care with patient. Hearing it from the patient’s mouth helps reduce - but not end - infighting and “what mom always wanted” opinion based arguments. Can also be had after HCP/POA/Will is executed as a fait accompli. 2. T4: Often challenging to engage with primary care providers PCPs while on active treatment but can be helpful. PCPs can help manage non-cancer care,offer preventive health such as vaccinations,discuss goals of care(esp if long-standing relationship) #CSSMChat #healthpromotion ¾ 3. Just to clarify: There is nothing morally wrong with CPR being withheld if this is consistent with a patient’s goals of care. To suggest otherwise and especially to imply that patient self determination at the end of life is evil is frankly irresponsible. 4. After a difficult Goals of care discussion with a family, a stranger standing close to the room (in plain clothes and no badge) stopped me to tell me that she was an attending from another hospital and that she admired my patience and communication skills during the encounter. | |
|  | Facilitating EOL Convos | Having the conversation; palliative care consults; convos; conversation –engaging in and facilitating it; expressing the need for it |
|  | 1. Usually these requests come from ICU teams...In my experience, there R often factors that I was unaware of that underpins the referral for RRT. Open discussion at Attg level can usually help for all parties to understand goals of care &amp; RRT 2. I don’t think I get many palliative consults anymore since covid hit tbh. Everything is goals of care and hospice evals. It’s sad because a majority of the consults are because treating MD just don’t want to even start the conversation. It’s basically a cold call these days. 3. #TipsForNewDocs Goals of care discussions are tough, &amp; even harder with restricted visitor policies. I ask “how frank would you like me to be?” Assess family readiness —&gt; ⬆️ productivity —&gt; better pt care. What do others do?! | |
|  | NHDD (Social Workers) | Social workers and social work efforts promoting NHDD |
|  | 1. April 16th is National Healthcare Decisions Day, which #NASW supports every year #NHDD inspires, educates &amp; empower providers &amp; ppl about the importance of advance care planning. #Socialworkers here is a guide on conversations about end of life planning. https://t.co/Q2FUZpde6k <https://t.co/3sZVC8MgIH> 2. Today is National Healthcare Decision Day. Living with a chronic illness means lots of healthcare decisions must be made. Get ahead of your decisions, empower yourself and make plans now to have all your legal paperwork in place and share with your circle what your wishes are. <https://t.co/qZt2kRRFRi> 3. We've announced a new resource to help people talk about end-of-life care. It's packed with info for patients, carers, families and professionals to help them record future wishes in the form of an advance care plan #wyhfuturewishes. Find out more https://t.co/RE1C2vOBV4 <https://t.co/09Gr7pyrU8> | |
| Surrounding Language | ACP Mistrust | ACP mistrust; events; challenges & promotion; biases in ACP, promotion & perceptions of ACP |
|  | 1. #JournalClub Recap: Landmark trial because (1) compares DOAC to placebo (2) includes a frail older population that was previously excluded from similar trials. As always, would depend on an individual's #goalsofcare but otherwise, promising data! #GeriMed #geriatrics 2. #PalliativeCare consultations have shown to reduce nonbeneficial, potentially inappropriate interventions, as decision for their use should always be made in the context of both the patient's prognosis &amp; the patient's #GoalsofCare https://t.co/GWnvkgYlHL #NPCW2021 3. Totally agree Down pointing backhand index Sometimes people mistakenly think DNR means “don’t do anything.” Remember, DNR only goes into effect when a patient codes. A DNR is not a substitute for a goals of care discussion. They are not the same thing. #MedTwitter #CriticalCare #GoalsOfCare 4. t happened again today- just now. a colleague who i work with was accused of racism because he dared speak to a family about goals of care for a patient who is dying on the vent. it is so upsetting that families throw racism out so casually anytime they are frustrated and angry | |
|  | Language Use in ACP | Language around ACP; proactive in filling out and continued ACP convos; process of ACP; Language in ACP; sarcasm; Dying Matters Week Awareness 2021 |
|  | 1. Are you keen to make sure you get the future care and treatment you’d want if you became too unwell to tell people your wishes? Lambeth residents can get free help to make an advance care plan: <https://t.co/urkVRNkWyY> 2. COVID-19 Goals of Care: Conversation Script: @vitaltalk conversation map including specific phrases to use when talking to patients who have COVID-19 about their goals of care &gt;&gt; https://t.co/ZsfF1tgZ9C #covid19 #pallicovid #healthcare 3. also, we should stop calling it “withdrawing care.” care is not withdrawn; our *goals* of care have changed. everyone deserves a dignified and comfortable death/passing on! <https://t.co/lyDgZklrO6> | |
|  | Involvement/Proxy | Involving others; naming a proxy; education; healthcare proxy; |
|  | 1. DYK? Everyone 18 years or older should have at minimum a Medical Durable Power of Attorney –– someone to make important medical decisions if you become unable to communicate your health care wishes. 2. Do you know what brings your loved one with an intellectual or developmental disability comfort if they fall ill? Use our NEW #COVID19 Advance Care Plan: Guide for Caregivers of Adults with #IDD to lead the conversation #ACP #AdvanceCarePlanning https://t.co/EivCB3rvuq https://t.co/vGALmvJZ4Y 3. Touchstone Life Care founder &amp; CEO Dr Merran Cooper shares how an Advance Care Plan started an important conversation between a mother and her son and why it offered the family invaluable piece of mind. | |
|  | Strategies for Providers | Approaches for HP to have ACP convos; quality of care; palliative care |
|  | 1. Like to increase your confidence in timely #GOC discussions? Our 'Planning #EndofLife Care - #GoalsofCare' free online learning can help you learn to &gt; discuss &amp; negotiate goals of care &gt; manage difference of opinion or expectations in care Learn more &gt; https://t.co/Bi3E7S47Ta <https://t.co/Wdbi6M09AT> 2. Palliative care consultation for patients with AKI in the ICU can improve patient’s quality of life and identify patients’ goals of care. The mnemonic PERSON may help facilitate communication in meetings with the patient/family. #PalliativeCareInNephrology https://t.co/xtLZwPMlHv <https://t.co/4pmwzul4Vx> 3. Auto anticoagulation should never be assumed and would not be considered protective for someone at risk of stroke risk. Bleed &amp; stroke risk are high, you need to discuss risk/benefits with the patient to discuss goals of care. Not easy. | |
|  | Narratives | Personal stories; doctor and patient communication; disease; illness barriers |
|  | 1. From a darling, deteriorating, elder contemplating goals of care “Tell me, sweet nurse, shall I die slowly or quickly?” I choked back my tears and replied “How about a goal of peacefully and let time do what it will.” Then we just sat in silence and held hands. #thosewecarry 2. What an absolute joy it was seeing family members at bedside today! Easier communication (putting a face to a name!), better advocacy for pts, easier goals of care conversations, better spirits and morale among patients and staff alike. 3. @JohnCSakles I recently had 90y/o severe AS with sepsis and ILD, lactate 12, in CHF. After I was convinced the goals of care had been thoroughly addressed (!) I did topicalized CMAC D Blade (“spray as you go”) with spontaneous breathing. No meds. No hemodynamic change. ðŸ | |
|  | Absence of ACP | Professional discussion about treatment vs. GOC discussions; doctor credentials; professional conversations; lacking; GOC/ACP missing |
|  | 1. I think like in most things #HaPC it really depends on the person, the situation, and the overall goals of care. Most of the review content with NIV was more of its use at EoL or when transitioning off a ventilator at EoL and if it was/would be useful/beneficial. 2. Some of my fave medical euphemisms: “Conservative management” = we’re not going to do anything “Goals of care discussions” = ahem they’re going to die “Functional” = fake “There isn’t evidence to support...” = LOL yeah we’re winging this shit What are urs #medtwitter 3. For some physicians, “goals of care” discussions has become shorthand for a hospice or #stopdoingwhatweredoing discussion. If this becomes the norm, pts/fams will resist engagement. #hpm #hapc <https://t.co/KHnFMHvgWV> | |
|  | Clarification Surrounding Terminology | Express how code status does not equal goals of care; defining GOC; need for communicating well |
|  | 1. ⛑️ Palliative care in R/R aggressive lymphomas:   - PC ≠ Hospice care or end-of-life care  - PC integration is low: too late and less than in solid tumors  - Identify triggers for goals of care discussions  - Collaboration between specialists  #ASH20 #ASHFromHome <https://t.co/p0fRVqsRdM>   1. RT @thelakesshow: Code status ≠ Goals of care   Goals of care ≠ Code status  Code status ≠ Goals of care  Goals of care ≠ Code status   1. Namaste, here is your unroll: @bumblebeenush: Excellent set of comics by Dr Jim Fulmer on how to approach “Goals of care conversations… https://t.co/XYSzr50TpX Have a good day. ð | |
|  | Importance of Having EOL Convos | How to have difficult conversations; not having conversations COVID-19 |
|  | 1. An important topic highlighting resident learner training needs on goals of care discussions at #SGIM21 @AsousSalma Tara Lieberman Sreekala Raghavan - great job Dr Asous! 2. diagnosed with stage III-IV cancer should have a palliative care appointment to define goals of care. We need to de-stigmatize this essential element of care. 3. At St John of God Health Care we respect and honour the dignity of each person we serve, and our commitment remains to care for all patients who require our service in line with our Mission. | |
|  | Goals of Care (GOC) | Homecare and identifying; promotion of tool to promote GOC; high risk patients; achieving goals and working with family |
|  | 1. A simple scoring tool to help stratify patients in terms of their medical ICU readmission risk based on patient and process factors. It can help facilitate close monitoring, early goals of care discussion, and proactive planning of high risk patients https://t.co/MjR9Dc3hNX <https://t.co/Xiq2Rlrnqf> 2. CONGRESS \| #ASH20 \| Oreofe Odejide, @DanaFarber, discussed 2 challenges. 1) Goals of care talks may come too late in patients with aggressive lymphoma. 2) Specialty palliative care integration is low and late for these patients. Possible solutions are shown in slides #lymsm <https://t.co/JnXuPhkX08> 3. Deb Barry, VP of Clinical Excellence &amp; Integrity at Intrepid USA, shares how WellSky's newest data analytics solution helps the company's clinical leaders understand each patient's specific needs, making it easier to achieve goals of care. #homehealth… https://t.co/6ijagcaWGQ | |
|  | Clinical/Biomedical Covid | COVID; ICU/hospitals; hospital; physician reports; stats/facts on hospital care surrounding ACP and end-of-life |
|  | 1. Friday. Goals of care conversation? Saturday. Suicide attempt? Sunday. Seeing majority of marginalized patients mistreated in our system? Daily. The system didn’t deserve half of me, let alone everything I had. The insult was the “meetings” with hire ups 3/7 2. The majority of people taking up the ICU beds are age 50-69. Out-and-about citizens. These are not the LTC folks. Most of the people in longterm care have goals of care declining ICU admission, or even hospital admission. They mostly die in their rooms in longterm care. 3. It’s definitely not the whole story throughout the pandemic. In the first two waves many people died without getting into the ICU because of their goals of care. But most of that group (usually &gt;80 y) has been fully vaccinated for months. Most in hospital deaths now are in ICU. | |
| Systemic Issues | Circumstances | Goals of care; relationships of context and circumstances to ACP |
|  | 1. Some elderly people may choose to remain in place for a disease such as this, but this must be their own choice. Let’s ensure robust #GoalsOfCare and #AdvancedHealthDirective discussions to ensure patients get the best care consistent with their preferences. Let’s not be callous. 2. I have such a love/hate relationship with breaking bad news conversations. They're so necessary but also really quite disappointing to not get direct positive feedback/response.#goalsofcare #breakingbadnews #MICUmonth 3. Goals-of-Care Consultations Are Associated with Lower Costs and Less Acute Care Use among Propensity-Matched Cohorts of African Americans and Whites with Serious Illness <https://t.co/8kIUKnktiE> 4. T1 - many barriers exist due to implicit bias. Lack of appropriate testing, follow-up, and assumptions about goals of care - all leading to poor outcomes #hcldr | |
|  | Approaches/Policy | Referrals; healthcare options; medical policy; approaches for ACP |
|  | 1. I learned from Dr. David Wu that goals of care convos are best done in a narrative fashion rather than a scripted algorithm that leads to the provider talking more. Approaching the convo w/ the pt story at the center ensures you know what matters most. Talk less, listen more. 2. This is so important, thank you for sharing! Reminds me of #covid19 #disparities in #mortality linked to differences in #GoalsOfCare across institutions. Those in centers w/less access to critical &amp; specialty care having greater chance of DNR, DNI, CMO orders. 3. 1 in 5 Americans is now serving in a caregiver role. There is no playbook for this taxing and complex role. @myeternallyio supports caregivers everywhere by letting the patient lead the discussion around their future goals of care @nytimeswell https://t.co/tjLTbaqx0s | |
|  | Discussions (Clinical) | Consults; interventions; drugs; ACP chatter; clinician conversations |
|  | 1. Keep sats 90 or higher, dex 6mg for 10d if o2 &lt; 92-94%, early GOC talk, chemical dvt ppx for all, remdes maybe?, if really considering abx check procal (only ~2% have bacterial CAP), keep net neg, trial proning, HFNC ?&gt; NIV, more goals of care :( 2. Can’t tolerate hemo. Mixed septic/cardiogenic shock. On the vent still as well. Things are going the wrong way so I’ve been advocating in rounds for goals of care talk and this still has yet to be done. 3. “The most common reason for ethics consultation among transplant candidates was discussion about intensity of treatment or goals of care after the patient was not or was no longer a transplant candidate “ @MGHMedicine⁩ | |
|  | Frailty & Covid | Frailty issues with GOC treatment; clinical COVID conversations; COVID advocates; long term care patients; duration of plan |
|  | 1. Also what is the number of LTC residents that died that are coded C1 or C2 for their goals of care designation. With this designation they provide comfort and no cure to those individuals for any illness. Flu, covid, heart attack, stroke, cancer. <https://t.co/SJsTWTu7h4> 2. I leave my goals of care at the door. My goal is to clarify values and set reasonable expectations. 3. Frailty is a key predictor of COVID-19 prognosis, and its assessment alongside measures of acute morbidity, rather than age… | |
|  | Programs | Care settings; highlighting disparity, cost and program needs |
|  | 1. Either decrease volumes, increase number of doctors/providers, and train these individuals for having effective goals of care conversations in hospital, or we need a MUCH more robust #palliativecare #hpm program in all hospitals, especially embedded in Geriatric, onc units, etc. 2. Because patients came to a subacute rehab actively dying, but nobody had sat down to have a meaningful conversation with the family. Nothing like having goals of care meeting when meeting family for first time when on call for the weekend. There’s hours, then heavy emotional load 3. Edsall et al. found that when it comes to critical decisions in the #trauma #ICU, goals of care #GOC conversations were primarily and effectively led by the TICU team, and this has been consistently increasing over the study period #palliativecare | |
|  | Healthcare Teamwork | Role of various healthcare parties in ACP/health exchanges; need for working together |
|  | 1. I certainly did. Fiona’s reading from her book placed a workable framework to hear from Simone with a Palliative Care perspective complemented with Li’s role as a Death Doula. Good insight for people planning ahead with their Advance Care Plan #LetsTalkAboutDeath @DyingMatters 2. Trying so hard! Knock on wood so far I would say every site I’ve worked at I/we avoided ER transfers while I’m on and still have had the site provide excellent care appropriate to goals of care. Plus the community paramedics are huge on keeping them home. It’s a team effort! 3. Understanding why some patients are treated and others are not seems challenging even when using the best propensity scores and will be a factor of how sick the patient looks, how much of an adopter the physician is, the goals of care of the patient, etc. | |
